# Do Children and Adolescents with Anorexia Nervosa Display an Inefficient Cognitive Processing Style?

**DOI:** 10.1371/journal.pone.0131724

**Published:** 2015-07-02

**Authors:** Katie Lang, Samantha Lloyd, Mizanur Khondoker, Mima Simic, Janet Treasure, Kate Tchanturia

**Affiliations:** 1 King’s College London, Psychological Medicine, Institute of Psychiatry, Psychology & Neuroscience, London, United Kingdom; 2 Biostatistics department, Institute of Psychiatry Psychology & Neuroscience, London, United Kingdom; 3 Child and Adolescent Eating Disorder Service, South London and Maudsley NHS Trust, London, United Kingdom; 4 Illia State University, Department of Psychology, Tbilisi, Georgia; Charité-Universitätsmedizin Berlin, Campus Benjamin Franklin, GERMANY

## Abstract

**Objective:**

This study aimed to examine neuropsychological processing in children and adolescents with Anorexia Nervosa (AN). The relationship of clinical and demographic variables to neuropsychological functioning within the AN group was also explored.

**Method:**

The performance of 41 children and adolescents with a diagnosis of AN were compared to 43 healthy control (HC) participants on a number of neuropsychological measures.

**Results:**

There were no differences in IQ between AN and HC groups. However, children and adolescents with AN displayed significantly more perseverative errors on the Wisconsin Card Sorting Test, and lower Style and Central Coherence scores on the Rey Osterrieth Complex Figure Test relative to HCs.

**Conclusion:**

Inefficient cognitive processing in the AN group was independent of clinical and demographic variables, suggesting it might represent an underlying trait for AN. The implications of these findings are discussed.

## Introduction

Anorexia Nervosa (AN) is an eating disorder (ED) characterised by restriction of calorie intake, problems with weight and shape [1], and carries an extremely high mortality rate [2]. Population-based studies have estimated prevalence rates ranging between 1.2–1.9%, and over recent decades there has been an increase in the incidence rates amongst young females (15–19 years), suggesting they represent a particularly high-risk group [3].

AN is also highly associated with obsessive compulsive, perfectionist and social communication difficulties, and such difficulties are postulated to be an intermediate phenotype triggered by a specific neurocognitive profile observed in adults with AN [4]. Inefficiencies with set-shifting (the ability to move flexibly between strategies, rules or behaviours,[5]) and weak central coherence (a bias towards details at the expense of global integration [6]), have been well established in the adult AN literature [7, 8]. These inefficiencies have been found to persist with recovery, with weight-restored individuals displaying an intermediate and attenuated profile [9]. There appears to be a genetic component to this cognitive profile, with it being observed in unaffected relatives of those with eating disorders, such as sisters and healthy offspring of mothers with AN [10, 11]. These findings have led to the postulation that this cognitive style is representative of an endophenotype for the disorder [9, 10, 12]. An endophenotype can be defined as a measureable component between a gene and the observable phenotypic characteristics of a disorder [13].

Neuroimaging studies in the adult AN population have identified structural differences in multiple brain regions in AN cohorts [14, 15]. More recently, functional differences have been identified in participants with AN when completing a central coherence task [16]. Such findings highlight the neural underpinnings that may be linked to the inefficient cognitive processing in AN.

There is evidence from healthy population studies that cognitive processing, and in particular mental flexibility, is important for wellbeing in general [17]. Furthermore it is evident that poor cognitive flexibility and a bias toward detail/poor global integration are risk factors for a range of psychiatric disorders [17], with Obsessive Compulsive Disorder (OCD) and Autism Spectrum Disorder being particularly associated with difficulties in these domains to varying degrees [18, 19]. However there is speculation as to whether such cognitive profiles are only markers for the OCD rather than being underlying endophenotypic traits. Unlike the adult AN literature, evidence of their stability appears to be inconsistent, with some studies showing improvements in cognitive processing following treatment [20, 21], suggesting they could be associated with the acute illness stage, as well as various clinical factors such as severity, symptom dimension and medication use [22, 23]. Results from the child and adolescent OCD studies appear to be conflicting, with one longitudinal study reporting improvement in cognitive processing with the remission of obsessive-compulsive symptomatology [24].

Neuropsychological processing in the child and adolescent AN population has been less widely studied than adult AN, which is surprising considering that neuroimaging studies have highlighted the presence of structural and functional abnormalities in younger AN populations [25].

The available data appears inconsistent, with some studies showing evidence of inefficient processing [26, 27], whilst others are unable to find differences between groups [28–31]. Such inconsistent findings may be due to the heterogeneous nature of the samples recruited in these studies. Systematic reviews of set-shifting and central coherence in children and adolescents with AN highlighted that small sample sizes and large variability in tasks used to assess cognition currently render the available data difficult to interpret [32, 33]. These reviews indicated that although non-significant, there was a trend toward both a more inflexible and a detailed focussed processing style amongst the child and adolescent AN groups. It is therefore clear that this is an avenue worth pursuing with further research, as it would be valuable to clarify the neurocognitive profile of children and adolescents with AN for a number of reasons.

Firstly, elucidating the neuropsychological profile of children and adolescents with AN would be advantageous in the development of more effective treatments for AN. For example, it will help to clarify whether remedial treatments such as Cognitive Remediation Therapy (CRT), which have been found to be effective in the adult AN population are likely to be beneficial in younger patients [34, 35].

Confirming whether or not children with AN show inefficient cognitive processing could contribute evidence to the endophenotype hypothesis and aid our understanding of the neurobiological and genetic underpinnings of AN. It could potentially be used as a marker for sub-typing ED’s and could help to discriminate AN from other EDs such other specified feeding and eating disorders.

Furthermore once the neuropsychological profile has been defined, this can be merged with our existing knowledge of structural and functional differences to strengthen our understanding of the neural mechanisms involved in AN.

With this in mind, the present study aimed to investigate neuropsychological processing in children and adolescents with AN by comparing their performance on a number of tasks to the performance of healthy controls. We hypothesise that children and adolescents with AN will show a more inflexible processing style and lower global processing abilities compared to their healthy control counterparts.

## Material and Methods

### Participants

Participants included children and adolescents with a diagnosis of AN (N = 41) and age, gender and IQ matched healthy control (HC) children and adolescents (N = 43). All participants were female. The age of the participants ranged from 11–18 years of age. This sample size was based on a power calculation for detecting a medium sized effect with 80% power and a level of significant of 0.01.

AN participants were recruited from the Child and Adolescent Eating Disorder Service, South London and Maudsley NHS Trust. Most were receiving either outpatient treatment, or were attending a residential day programme. Two participants were receiving inpatient treatment. The inclusion criteria for the AN group included a DSM-5 diagnosis of Anorexia Nervosa and Ideal body weight (%IBW) of 90% or under. Percentage IBW shows what proportion of their optimal body weight an individual currently is, and it is corrected for age and gender [36].

Inclusion criteria for the HC group included a %IBW of 91% or above. Participants with a %IBW of 120 or over were excluded from the study. Participants were also excluded if they had a current or past history of an eating disorder or any other psychiatric disorder. This information was obtained during the SCID interview. Participants were also excluded if there was a current or past history of and ED in first or second degree relative. This information was collected with the demographic information. Exclusion criteria for both groups were a diagnosis autism spectrum disorder (ASD), psychosis, learning difficulty or being non-fluent in the English language. Within the AN group 85.4% were White British, and 81.4% of the HC group were White British.

All participants signed consent forms, and parental consent was also gained for each participant. This research was approved by a Research and Ethics Committee (REC no: 12/LO/2015).

### Measures

#### Demographic measures

Percentage Ideal body weight (%IBW): A percentage of each participant’s ideal body weight was calculated from measurements of their weight and height and corrected for age and gender [36].

The Structured Clinical Interview for DSM-IV Axis I disorders (SCID-I, [37]): Was used as a screening tool to assess the presence of possible psychiatric disorders in the AN and HC groups.

#### Neuropsychological measures

Intelligence Quotient (IQ): Weschler Abbreviated Scale of Intelligence (WASI [38]): The WASI is formed of the four subscales block design, vocabulary, matrix reasoning and similarities. Raw scores are calculated for each subscale and then scores are scaled by correcting for age. Scaled scores are then summed to give the verbal, performance and full scale IQ scores.

Set-shifting: The Wisconsin Card Sorting Test Computer version 4 (WCST[39]): Measure of set-shifting. Participants must match a number of stimulus cards to one of four category cards. Cards can be matched by colour, number or shape, and the rule must be worked out by trial and error based upon the feedback received. Once the participant has correctly matched the card for 10 consecutive sorts, the sorting rule changes and the participant must shift their response to work out the new sorting rule. The rule changes up to five times throughout the task, and every time a participant correctly completes a sort this is termed ‘completing a set’.

The most commonly reported outcome from the WCST is the number of perseverative errors made by the participant. However, in order to mimic the largest dataset in adult AN [7], the current study will also report measures of general performance, perseveration, conceptual ability and response consistency. Further descriptions of each of these measures can be found in Tchanturia et al., 2012. The present study replicated the test parameters of Tchanturia et al., (2012), where by visual and audio feedback is given in a male voice one second after each sort has been made.

Central Coherence: The Rey-Osterrieth Complex Figure test (ROCFT[40]): A pen and paper task measuring global processing ability, participants are required to accurately copy a complex figure and the drawing strategy adopted by the participant is used as a measure of central coherence. The ROCFT is scored according to Booth’s 2008 scoring method [6], which incorporates both the order in which the participant chooses to draw the elements (whether preference is shown to global or detailed elements) and the style in which they are drawn in (fragmented or coherently). Order index (OI) and style indexes (SI) are computed and are added to give the Central Coherence Index (CCI). For more details see Lopez et al., 2008 [41]. In order to check reliability, 15% of the entire sample was co-rated by a second rater. Inter-rater reliability was good with Cohens kappa = 0.71, p = < .001.

The Fragmented Pictures Task (FPT, [42]): A computerised task in which participants are presented with a picture that slowly develops frame-by-frame. Participants are asked to indicate verbally what the object is as quickly as possible. There is one practice trial followed by six test trials. The main outcome measure is the mean frame in which the participant correctly identifies the picture. The task is benefited from a global approach and quicker responses are indicative of better global processing [8, 42].

Self-report Questionnaire measures: Eating Disorder Examination Questionnaire (EDEQ [43]): A 36-item scale assessing eating disorder psychopathology. Clinical criteria cut-off adopted was a global score of higher than 2.7[44, 45]. Cronbach’s Alpha for the current study was 0.98.

Hospital anxiety and depression scale (HADS [46]): Measures anxiety and depression symptomatology. The clinical cut off for this measure is 10. Cronbach’s Alpha for the current study was 0.91.

Child Obsessive Compulsive Inventory (Choci [47]): Used to assess obsessive-compulsive symptomology in children and adolescents. The measure assesses both the content and the severity of the symptoms with a total score is calculated out of 40. Scores of 17 or above are suggested as a clinical threshold. Cronbach’s Alpha for the current study was 0.94.

Autism-Quotient 10 (AQ-10, [48]): A shortened version of the Autism Quotient, used to assess autistic features and traits. The clinical cut-off is six or above. Cronbach’s Alpha for the current study was 0.54.

The Detail and Flexibility questionnaire (DFLEX,[49]): The DFLEX is a 24-item scale assessing cognitive rigidity/detail focussed processing. Cronbach’s Alpha for the current study was 0.80.

### Procedure

Participant’s height and weight data were collected on the morning of testing using SECA electronic scales, by a clinician as part of a treatment programme. Participants were asked to complete the self-report questionnaires and then the neuropsychological tasks were administered by a trained researcher (KL/SL).

### Statistical analysis

Histograms were used to assess the data for normality. A number of the WCST outcomes were positively skewed (total response errors, perseverative responses, perseverative errors, non-perseverative errors, failure to maintain set). Standard log transformations were conducted to normalise the data. T-tests were used to test for differences in performance between the two groups on the transformed data. Log transformations were unable to normalise the learning to learn scale of the WCST, and so a Mann Whitney-U test was used. Due to the testing of multiple variables, we used a more conservative significance level of 1%, to minimise Type I errors.

The effect of confounding variables such as age and IQ were tested for and were non-significant and so these were not included in the final statistical model.

Correlations were used to examine associations between neuropsychological performance and clinical and demographic characteristics within the AN group. Cohen’s d was calculated to provide effect sizes (<0.2 = small, <0.5 = medium, >0.8 = large). Data was analysed using the statistical package IBM SPSS version 21.00.

## Results

Clinical and demographic data for the participants is displayed in Table 1.

**Table 1:** Participant demographics table (Means & SD).

	AN (N = 41)	HC (N = 43)	P value	Effect size (Cohen’s d)
**Age (years)**	15.07 (1.81)	15.11 (1.94)	.93	-
**%IBW**	80.68 (6.57)	101.1 (8.41)	< .001(***)	-
**BMI**	16.16 (1.50)	20.34 (1.99)	< .001 (***)	-
**Years of education**	11.05 (2.00)	10.86 (1.90)	.77	-
**Illness duration (Years)**	1.70 (1.18)	-	-	-
**Medication Use (%)**	51.20	-	-	-
**SSRI (%)**	38.10	-	-	-
**Olanzipine (%)**	38.10	-	-	-
**Both (%)**	23.80	-	-	-
**Age of onset (Years)**	13.80 (2.30)	-	-	-
**EDEQ Global**	3.45 (1.79)	0.99 (0.70)	< .001(***)	1.85
**AQ-10**	3.90 (2.12)	2.67 (1.27)	< .01(**)	0.72
**HADS Anxiety**	12.34 (5.05)	4.72 (2.49)	< .001(***)	1.95
**HADS Depression**	7.93 (4.46)	2.09 (1.59)	< .001(***)	1.83
**Choci**	14.55 (13.11)	3.89 (5.32)	< .001(***)	1.09
**DFlex Cog ridgidity**	50.17 (14.62)	34.79 (7.40)	< .001(***)	1.35
**DFlex Attention to detail**	45.51 (14.62)	36.31 (7.89)	< .001(***)	0.80

Footnotes:

* Significant at 0.05

**0.01

***0.001

%IBW = %ideal body weight; BMI = body mass index (kg/m^2^); EDEQ = Eating disorder examination questionnaire; AQ10 = Autism Quotient 10; HADS = Hospital anxiety and depression scale; DFlex = Detail and flexibility scale; Choci = Child Obsessive Compulsive Inventory.

### IQ

There were no significant differences between the AN group and the HC group on any of the scales or subscales. However, there was a trend for higher performance in the AN group on the matrix reasoning sub-scale (see Table 2).

**Table 2:** Means (SD) and p-values for WASI.

	AN (N = 41)	HC (N = 43)	P value	Effect size (Cohen’s d)
**Verbal IQ**	105.92 (21.30)	105.63 (11.26)	.94	0.02
**Performance IQ**	103.55 (14.00)	104.07 (11.81)	.86	0.04
**Full Scale IQ**	107.10 (13.76)	105.30 (10.29)	.50	0.15
**Vocabulary**	58.13 (10.02)	55.29 (8.23)	.23	0.31
**Similarities**	53.00 (16.12)	48.35 (6.34)	.14	0.39
**Block design**	56.31 (9.28)	52.32 (8.70)	.10	0.47
**Matrix reasoning**	56.31 (9.28)	51.94 (7.44)	< .05(*)	0.53

Footnotes:

* Significant at 0.05

**0.01

***0.001

IQ = Intelligence quotient

### Neuropsychological outcomes: Set-shifting and central coherence

**Fig 1:**
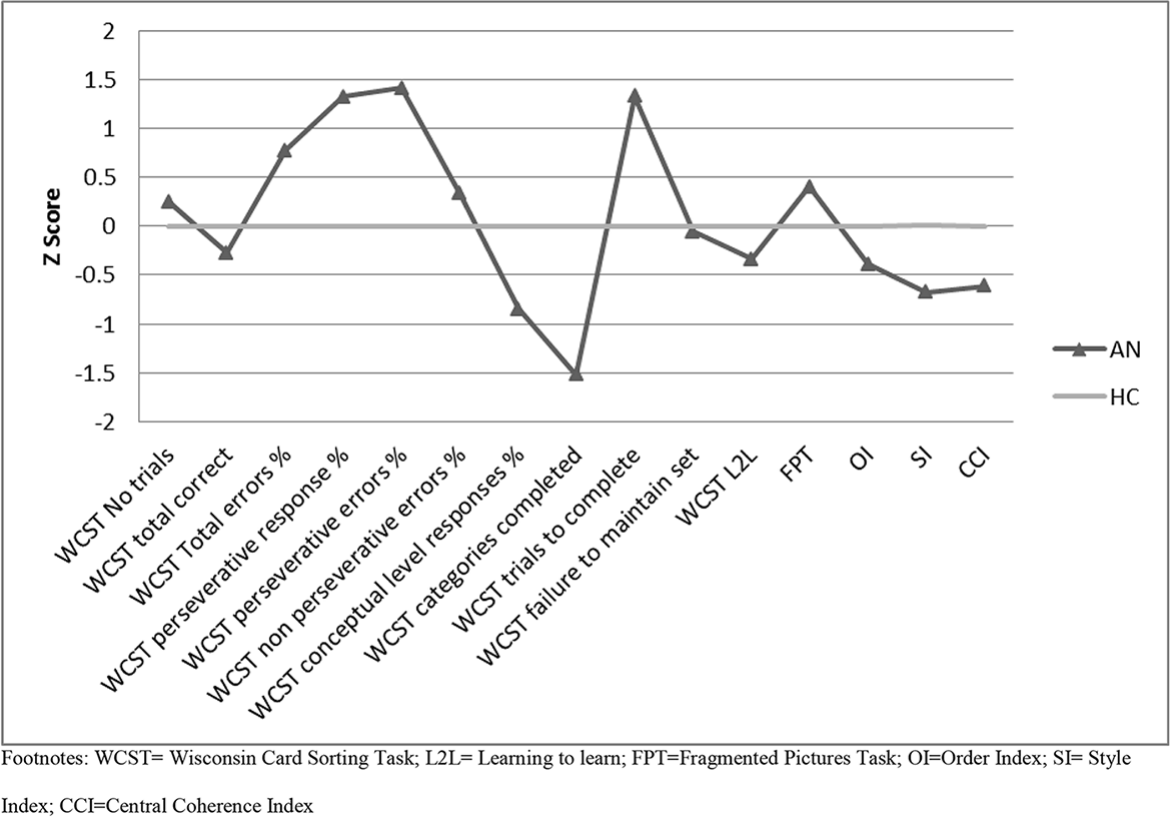
Z scores for neuropsychological performance for AN and HC groups. Footnotes: WCST = Wisconsin Card Sorting Task; L2L = Learning to learn; FPT = Fragmented Pictures Task; OI = Order Index; SI = Style Index; CCI = Central Coherence Index.

Fig 1 displays the z-scores for each group for the neuropsychological measures. Z-scores were calculated using the formula: (data point–healthy control mean)/healthy control SD, to depict the cognitive profile of the AN group against that of the HC group [5].

The figure suggests that there are no impairments in the AN group on any of the tasks (no scores fall below -1.5/-2), however the differences in processing style between the groups can be seen.

#### Wisconsin card sorting test

Table 3 displays the transformed and untransformed means and standard deviations for each of the WCST outcomes with p values and effects sizes.

**Table 3:** Set-shifting and central coherence outcomes: Means (SD) or medians (IQR), p-values and effect sizes.

Set-shifting outcomes: Wisconsin Card Sorting Test				
	AN (n = 41) Mean (SD)/ Median (IQR)	HC (n = 43) Mean (SD) Median (IQR)	P value	Effect size (Cohen’s d)
*General performance measures*				
Number of trials administered	95.7 (20.2)	91.5 (17.1)	.28	0.23
Total correct responses	69.8 (11.1)	72.4 (9.5)	.62	0.00
Total response errors	26.2 (22.5)	18.3 (9.9)	-	
Total response errors %	18.0 (14.0–29.0)	18.5 (15.0–23.0)		
Total response errors% (transformed)	3.0 (0.5)	2.9 (0.3)	.25	0.25
Total categories completed	5.4 (1.4)	5.9 (0.3)	< .05(*)	0.51
*Perseveration measures*				
Perseverative responses	14.4 (15.1)	8.6 (4.6)	-	
Perseverative responses %	10.0 (8.0–15.5)	8.5 (7.0–10.3)		
Perseverative errors	13.1 (12.4)	8.1 (3.9)	-	
Perseverative errors %	10.0 (8.0–13.0)	8.0 (6.75–9.25)		
Perseverative errors% (transformed)	2.3 (0.5)	2.1 (0.3)	< .01(**)	0.49
Non-perseverative errors	13.2 (12.9)	10.2 (6.6)		
Non-perseverative errors %	9.0 (6.5–9.0)	10.0 (7.0–13.0)		
Non-perseverative errors% (transformed)	2.3 (0.7)	2.3 (0.5)	.81	0.00
*Conceptual ability measures*				
Trials to complete first category	16.8 (20.1)	12.5 (3.2)	.43	0.31
Conceptual level responses	63.3 (15.0)	68.7 (8.6)	.28	0.45
Conceptual level responses %	69.4 (20.5)	76.9 (8.9)	-	
*Response Consistency measures*				
Failure to maintain set	0.0 (0.0–1.0)	0.0 (0.0–1.0)	-	
Failure to maintain set (transformed)	0.9 (0.3)	0.9 (0.3)	.98	
Learning to learn	-1.26 (−2.7- −1.3)	-1.0 (−2.9- −1.0)	.26	
Central Coherence outcomes: Rey Osterrieth Complex Figure Test & Fragmented Pictures Task				
** **	AN (n = 41)	HC (n = 43)	Test statistic	Effect size (Cohen d-s)
FPT (Mean frame)	4.6 (0.7)	4.3 (0.6)	.09	0.38
ROCFT Order Index	1.5 (0.7)	1.8 (0.6)	.10	0.36
ROCFT Style Index	1.2 (0.5)	1.4 (0.4)	< .01(**)	0.62
ROCFT Central Coherence Index	1.0 (0.4)	1.3 (0.4)	< .01(**)	0.57

Footnotes:

* Significant at 0.05

**0.01

***0.001

IQR = Interquartile ranges; FPT = Fragmented Pictures Task; ROCFT = Rey Osterrieth Complex Figures Test.

There were significantly more perseverative errors (p = < .01) and perseverative responses (p = < .01) made by the AN group, and there were trends towards significant differences in the number of categories completed (p = .03).

#### Rey-Osterrieth Complex Figure Test and Fragmented Pictures Task

Table 3 displays the means and standard deviations for each of the central coherence measures. The AN group showed significantly lower scores on the style index (p = < .01) and the central coherence index (p = < .01) of the ROCFT. There was a trend for the AN group to show higher scores on the FPT (p = .09), indicating a more detail focussed approach.

### Sub-analyses within the AN group

#### Medication analysis

To assess the impact of medication on neuropsychological processing the AN group was divided into two sub-groups 1) medication free and 2) on medication, and a t-test was used to assess any differences in neuropsychological processing between the two groups in perseverative errors in the WCST, ROCFT and FPT. There were no significant differences between the groups on any of the neuropsychological measures (WCST perseveration errors p = .94, Order Index p = .88, Style Index p = .64, CCI p = .87, FPT p = .77).

#### Associations with clinical and self-report measures

Correlations were used to test for associations between the neuropsychological performance measures (WCST perseveration errors, SI, OI, CCI and FPT) and clinical (%IBW, age of onset, length of illness) and self-report data (autistic traits, depression, anxiety and obsessive-compulsive traits). There were no associations between the WCST, OI, SI or CCI and the clinical or demographic variables. However there were some weak associations between the FPT and EDE-Q Global scale (.27, p = .09).

## Discussion

This study aimed to investigate the neuropsychological processing profile of children and adolescents with AN. The findings suggest that they share a similar profile to adults with AN, with marked cognitive inefficiencies in set-shifting and central coherence relative to IQ. Firstly we found that there were no significant differences in any of the IQ scales or subscales of the WASI, with both groups exhibiting the same intelligence profile. Such findings are in accordance with previous studies of IQ in childhood AN, which also reported no differences in performance in comparison to HCs [50].

Regarding the neuropsychological measures we found that there were a significantly higher number of perseverative errors made by the AN group in the WCST, of medium effect size (d = 0.49). The AN group also exhibited lower style index and central coherence index scores on the ROCFT with medium effect sizes (d = 0.62 and 0.57 respectively). As can be seen from the Z-scores in Fig 1, the performance of the AN group in these task cannot be described as ‘impaired’, however, the AN group display a significantly different processing style to the HC group, characterised by subtle inefficiencies in both set-shifting and central coherence tasks. Overall, despite having similar IQ to the HC group, these results suggest an inflexible thinking style with poorer global integration in the AN group in young people. This is the first study to demonstrate such inefficiencies in children and adolescents using the WCST and ROCFT, and mirror the effect sizes found in large experimental studies in the adult AN and HC females using the same tasks and same test parameters and scoring methods [7, 8]. However, it should be noted that unlike the results from adult AN studies [7], whereby there were differences between AN and HCs across all domains of the WCST, the present study only observed worse performance by the AN group in perseverative errors and perseverative responses. Such findings could suggest that domains other than perseveration could be more susceptible to the effects of starvation and chronicity of ED pathology, as theoretically the participants in adult AN studies are likely to have a longer duration of illness.

Secondary analyses within the AN group demonstrated that cognitive inefficiencies were independent of medication status and other clinical and demographic variables, such as %IBW, illness length or anxiety, depression and OCD symptomatology.

Systematic reviews and meta-analyses of set-shifting and central coherence in children and adolescents with AN found non-significant but worse performance within the AN group compared to HCs. It was highlighted that such non-significance could be due to small sample sizes and differences in methodology [32, 33]. The findings of the present study in part support this idea, as it had a large sample size, used robust neuropsychological tasks with the same test parameters of the adult studies and found significant differences between the AN and HC groups.

These findings add support to the possibility that the cognitive inefficiencies observed in this study are underlying traits. This is further supported by the fact the AN group were in the very early stages of illness. In studies with shorter durations of illness the effects of starvation or consequences of illness on cognitive processing should be less pronounced leaving underlying traits more visible. Although the presence of these characteristics may add support to the idea that they may be an endophenotypic trait for AN, they cannot confirm the hypothesis. Particularly as one recent study demonstrated that cognitive processing improved and was comparable to HCs, following weight restoration [51]. Further studies using long-term weight restored children and adolescents are required in order to confirm this.

Our findings carry important clinical and research implications. Firstly, the clarification that children and adolescents with AN do share the same cognitive processing inefficiencies as adults with AN has important implications for the treatment of AN. Although a large proportion of children and adolescents respond well to outpatient treatment (family therapy having the most efficacy [52]), there is still a significant amount of patients who do not appear to benefit from this treatment [53]. This suggests that the field needs to continue to develop effective treatments for the child and adolescent AN age group.

The observation of this specific neuropsychological profile in adults with AN lead to the development of Cognitive Remediation Therapy (CRT) for ED [54]. CRT is proving a popular and beneficial treatment for adults with AN, and there is emerging evidence demonstrating improvement to cognitive processing styles in adults [35]. There is already emerging evidence that young people find CRT a beneficial addition to treatment [55–57]; however it is most commonly used with the severe and enduring population to minimise discomfort. The evidence of a similar profile in adolescents strongly suggests that CRT could be a beneficial treatment for younger patients with AN, to help remediate inefficient cognitive processing.

Adding CRT to treatment protocols may be associated with improved outcomes for children and adolescents with AN, however, further randomised controlled trials of CRT with children and adolescents with AN are needed to confirm this.

Taken together with the findings of inefficient cognitive processing persisting following recovery and weight restoration in adults, this specific cognitive profile could be used to sub-type AN from other eating disorders. Further work examining the neuropsychological profile of other sub-types of eating disorders such as bulimia nervosa (BN) and binge eating disorder (BED) are needed.

The strengths of this study lie in its large sample size and the use of the same robust neuropsychological tasks that are used in the adult AN literature. A further strength is that it replicates the methodology used in the largest adult AN neuropsychological studies, allowing for comparisons with published data.

A methodological limitation of the present study is that pre-morbid weights were not reported. Therefore the degree of weight loss and the severity of starvation of each participant cannot be accounted for in the results. It is important for future studies to collect this data and account for it in the analyses.

Although this study’s findings suggest underlying cognitive inefficiencies are present in children and adolescents with AN, it is important to acknowledge the typical developmental trajectory of both set-shifting and central coherence abilities. As discussed in a systematic review of set-shifting in children and adolescents with AN [32], previous research with healthy population samples has clarified that set-shifting abilities rapidly develop up to the age of eight years, with a moderate amount of development of these skills in early adolescence [58]. In terms of central coherence, there is a lack of developmental studies available, and it is suggested that central coherence can be viewed more as a processing style rather than an impairment or skill, and is based on a continuum from detailed-focussed to global integration [59]. The effects of starvation and malnutrition on the brain at such a critical developmental period should not be underestimated and should be considered when interpreting our results.

With this in mind, it will be important for future research in this area to use longitudinal designs and also to utilise neuroimaging to confirm whether the structural differences observed in adults samples are the same in younger populations. This will also help to identify the possible neural underpinnings of such inefficiencies.

The present study incorporated both the ROCFT and FPT to assess global processing. As performance on both of these tasks is benefitted from taking a more global approach, it would be beneficial for future studies to also use tasks where a detailed-focussed processing style is preferable, such as the group-embedded figures task [60]. This would allow us to confirm if children and adolescents with AN display weak central coherence (both a bias towards detail at the expense of the bigger picture), as is seen in the adult population [61].

To summarise, the present study has highlighted that children and adolescents with AN are likely to display a similar cognitive processing style to adults with AN, relative to IQ. These cognitive processing inefficiencies appear to be independent of clinical and demographic variables, suggesting that they could represent an underlying trait. Such findings carry important implications for the diagnosis and treatment of children and adolescents with AN.
